# Land use mix and five-year mortality in later life: Results from the Cognitive Function and Ageing Study

**DOI:** 10.1016/j.healthplace.2015.12.002

**Published:** 2016-03

**Authors:** Yu-Tzu Wu, A. Matthew Prina, Andy Jones, Linda E. Barnes, Fiona E. Matthews, Carol Brayne, MRC CFAS

**Affiliations:** a Department of Public Health and Primary Care, Institute of Public Health, Forvie Site, University of Cambridge, School of Clinical Medicine, Cambridge Biomedical Campus, Cambridge CB2 0SR, United Kingdom; b King's College London, Institute of Psychiatry, Psychology and Neuroscience, Centre for Global Mental Health, Health Service and Population Research Department, De Crespigny Park, Denmark Hill, London SE5 8AF, United Kingdom; c Norwich Medical School, University of East Anglia, Norwich, Norfolk NR4 7TJ, United Kingdom; d MRC Biostatistics Unit, Institute of Public Health, University of Cambridge, Cambridge CB2 0SR, United Kingdom; e Medical Research Council Cognitive Function and Ageing Study, United Kingdom

**Keywords:** Land use mix, Neighbourhood, Mortality, Older people

## Abstract

This study explores the potential modifying effect of age and mediation effect of co-morbidity on the association between land use mix, a measure of neighbourhood walkability, and five-year mortality among the 2424 individuals participating in the year-10 follow-up of the Cognitive Function and Ageing Study in England. Postcodes of participants were mapped onto Lower-layer Super Output Areas, a small area level geographical unit in the UK, and linked to Generalised Land Use data. Cox regression models were fitted to investigate the association. For the younger older age group (75–79 years), the effect of high land use mix on an elevated risk of mortality was mediated by co-morbidity. For older old age groups (80–84, 85+ years), a higher land use mix was directly associated with a 10% lower risk of five-year mortality. The findings suggest differential impacts of land use mix on the health of the younger and older old.

## 1 Introduction

In the UK, it is estimated that older people spend over 80% of the time in home or surrounding neighbourhoods (Age UK, 2015, Phillipson, 2012) and they largely rely on local services and resources such as post offices, banks, supermarkets and parks (International Longevity Centre UK, 2014, Harrop and Jopling, 2009). Access to these services has been examined in a recent analysis of the English Longitudinal Study of Ageing (ELSA), which highlighted difficulties in travelling far to a range of key services (banks, hospitals, post offices and supermarkets) for those aged 80 or above due the difficulties of obtaining transport (Holley-Moore and Creighton, 2015). Since walking and the use of public transport are the principal modes of travel in older age (Help the Aged, 2008, Holland et al., 2005), providing a supportive environment with nearby services may have mobility and consequent health benefits in this population (Holley-Moore and Creighton, 2015).

A range of environmental characteristics in local areas are thought to be important for active ageing and good health in later life (Annear et al., 2012, Yen et al., 2009, World Health Organisation, 2002). In particular, the diversity of land uses has been identified as being related to physical activity and mobility in older adults (Van Cauwenberg et al., 2011, Rosso et al., 2011; Li et al., 2005). Areas with high levels of land use mix generally indicate better access to local services and resources and have been shown to increase capabilities of older people to cope with basic needs (Rosso et al., 2013), encourage outdoor activity (Clarke and Nieuwenhuijsen, 2009) and enhance social engagement (Leyden, 2003) with potential benefits on physical and mental health in later life.

**Fig. 1:**
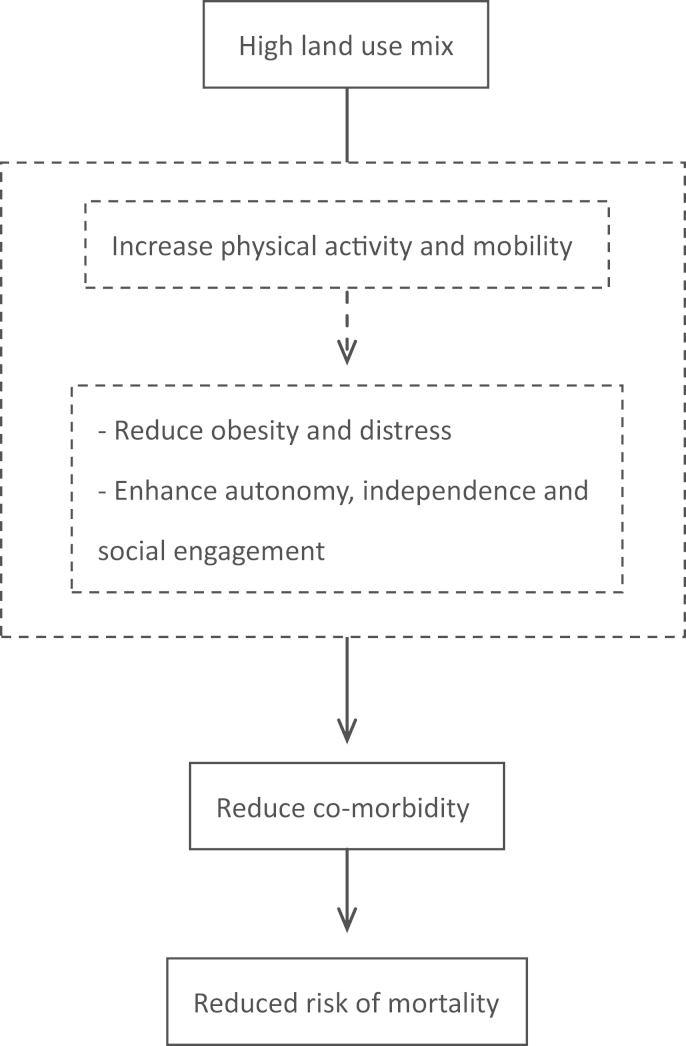
A conceptual framework of pathways linking land use mix and mortality. Dashed line: this study does not address these factors.

The conceptual framework in Fig. 1 shows potential pathways linking land use mix to mortality. High land use mix is known to be a protective factor for physical inactivity and obesity (Jones et al., 2007, Mackenbach et al., 2014), which have in turn been related to several chronic conditions such as diabetes, hypertension and cardiovascular diseases (Durstine et al., 2013, World Health Organisation, 2015). Being able to access services and cope with basic needs may also enhance independent living and social interactions (Holland et al., 2005), with potential psychosocial effects on mental well-being, quality of life as well as the development of chronic conditions (Almedom, 2005, Bowling, 2005, Hawkley and Cacioppo, 2003). These behavioural and psychological factors might influence the occurrence of co-morbidity, which is a strong predictor for reduced life expectancy and mortality (Lozano et al., 2012) and a potential mediator in the association between land use mix and mortality.

Most of the existing studies on older people have actually focused on “young old” those aged 60–74 years old with relatively good health, functional ability and social engagement compared to the “middle old” (75–84) and “oldest old” (85+) (Zizza et al., 2009). Despite showing resilience through survival, the middle and oldest old are more likely to experience frailty and illness (Baltes and Smith, 2003, Tomassini, 2006) and can be sensitive to stress from local environments (Lawton and Nahemow, 1973). For example, areas with mixed commercial, industrial and residential land use are often situated in the inner-urban core and their residents may be exposed to higher levels of common urban stresses such as social disorder, noise and concentrated poverty (Grant et al., 2009; Wikström et al., 2012). Indeed, a small number of studies have reported a potential negative effect of high land use mix and environmental stress, particularly on the mental health in older people (Saarloos et al., 2011, Knipscheer et al., 2000). However, few studies have explored changing relationships between the environment and health at different stages of ageing.

In order to provide a better understanding of ageing and place, this study aims to investigate the influence of land use mix on five-year mortality in a very old population cohort aged 75 and over in England. The analysis explores the potential modifying effect of age and mediation effect of co-morbidity on the longitudinal association between land use mix and mortality.

## 2 Method

### 2.1 Study population

The Medical Research Council (MRC) Cognitive Function and Ageing Study (CFAS) is a longitudinal population-based study investigating the cognitive and physical decline of people aged 65 and over in six centres across England and Wales (Liverpool, Cambridgeshire, Gwynedd, Newcastle upon Tyne, Nottingham and Oxford). Identical study designs and measurement methods were used at each centre except Liverpool, which is excluded from many CFAS analyses as well as the work presented here.

Full details of CFAS have been described elsewhere (Brayne et al., 2006). In brief, community and institutionalised populations were sampled from General Practice Registers in order to capture equal sized samples of those aged 65–74 and 75 years and over. Baseline interviews were conducted between 1991 and 1994 and delivered by trained interviewers visiting participants' residences. Among the 16,258 individuals invited for the study, 13,004 completed the initial screening interview with a response rate of 80%. The follow-up wave was conducted after 2 years from the baseline and then focused on sub-samples every two years after the 2 year with a 10 year follow-up on all survivors and responders.

Due to limited environmental data from the 1990s, this study focused on the 10 year follow-up in 2001. As comparable environmental data at the small area level are not available for Wales, the four identical English centres (Cambridgeshire, Newcastle upon Tyne, Nottingham and Oxford) were used in this analysis.

### 2.2 Individual level measurements

Mortality was the outcome of interest in this study. Date of death for the CFAS participants was available from linkage to national death certification. The information was used to identify deaths within five years from the year-10 interview (i.e. from the beginning of 2001 to the end of 2005) and calculate survival time. The choice of five-year endpoint was based on the consideration that exposure to environmental characteristics may vary significantly over longer time periods and hence relationships with mortality could be obscured.

Individual socio-demographic factors including age, gender, education and social class have been shown to be consistently related to general health and mortality risk and may confound associations with land use mix (Marmot et al., 1991, Tiainen et al., 2013, Kulhánová et al., 2014). Age was categorised into three groups: 75–79, 80–84 and 85 or above. Education was divided into “high” and “low” groups based on the CFAS study protocol which differentiated people with nine or fewer years of education from those with ten years or above (Brayne et al., 2006). The lifetime longest occupation reported in was used to classify social class of each participant according to the Registrar General's occupation-based social class (Office for National Staistics, 1990). The participants were then grouped into four groups: professional/managerial (social class I and II), skilled non-manual (IIINM), skilled manual (IIIM) and semi-skilled/unskilled (IV and V).

Co-morbidity was assumed to be a mediator on the longitudinal association between land use mix and mortality. The measure of co-morbidity was generated based on self-reported information on chronic conditions in the year-10 interview and divided into two groups: individuals with none or one chronic condition and those with two or more. Since those who had moved in the past two years would not have been exposed to their local environments for long, the interview question “have you moved in the last two years?” was used to identify those who had moved in that period in order to control for the potential influence of relocation.

### 2.3 Area level factors

Using information from the National Statistics Postcode Directory (NSPD) (Office for National Statistics), postcodes of the year-10 participants were mapped to Lower-layer Super Output Areas (LSOA), a geographical unit developed for the collation of small area statistics in the UK Census, with an average of 1500 residents per unit. The land use data for each LSOA were based on the Generalised Land Use 2001 dataset, which was obtained from the Neighbourhood Statistics database (www.neighbourhood.statistics.gov.uk), a collection of small area level data across England. The measure of land use mix was set to indicate the diversity of land use types in each LSOA and the calculation method was based on the existing literature with a range from 0 (lowest mix of land use) to 1 (highest) (Frank et al., 2006). Using the distribution of the whole population, the measure of land use mix was divided into quartiles using the lowest quartile as the reference group.

Area deprivation has been known to be associated with high risk of mortality (Meijer et al., 2012) and is generally related to high levels of land use mix (Grant et al., 2009). It has been suggested to be a neighbourhood level confounding factor (Chaix et al., 2010) and therefore was adjusted for to examine the independent influence of land use mix on mortality. In this study, deprivation scores were measured by the English Indices of Multiple Deprivation (IMD 2004), which was derived based on data collected in 2001 and 2002 (Neighbourhood Renew Unit, 2004).

### 2.4 Analysis strategy

The analysis first examined the unadjusted relationship between five-year mortality, land use mix and covariates. Cox proportional hazards regression was used to estimate unadjusted and adjusted hazard ratios in the whole population. Variables used for the adjustment included age groups, sex, education, social class, relocation and deprivation scores. Tests for trend were used to examine how the risk of mortality changed across the quartiles of land use mix.

To explore the modified effect of age, Kaplan–Meier failure curves of cumulative incidence of five-year mortality were estimated by the quartile of land use mix and further stratified by the three age groups (75–79, 80–84 and 85+) to explore the potential modified effect of age on the association. The effect sizes of land use mix on five-year mortality were estimated using proportional hazard regression. Interaction terms between the three age groups and land use mix were used to investigate whether the associations significantly differed by age and as a result the models were stratified by three age groups.

**Fig. 2:**
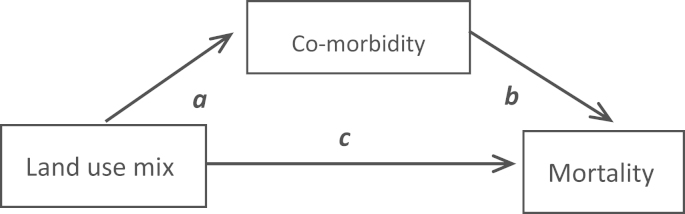
The association between mortality and land use mix and mediation effect of co-morbidity.

To investigate the potential mediation effect of co-morbidity on the association between land use mix and mortality (Fig. 2), this study adapted the approach recommended by Zhao et al. (2010). Using this method, two models were fitted to estimate the indirect and direct effects. First, a logistic regression was fitted to examine the association between comorbidity and land use mix, whereby the measure of land use mix was regressed on the dichotomous measure of comorbidity with adjustment for individual level factors and deprivation scores. Second, the longitudinal association between co-morbidity and mortality and the direct effect of land use mix on mortality were estimated by a proportional hazard model including all individual level factors, deprivation scores, land use mix and the measure of co-morbidity. In order to provide summarised and stable estimates of effects, the mediation analysis focused on trends across quartiles of land use mix and estimated changes in mortality risk per increased quartile. These models were stratified by the three age groups. Statistical significance was defined based on 95% confidence intervals around hazard ratios/odds ratios (excluded 1.0) or *p*-values (less than 0.05).

## 3 Results

The characteristics of the 2424 participants in this analysis are presented in Table 1. The median age was 81 with a range from 75 to 102 and over one-quarter (27%) were aged 85 or over. More women (61%) were included in this study population, as would be expected based on the cohort age. About 15% of participants (*N*=364) moved in the past two years. Cumulative mortality was 24.7% at five years. The mortality rate before 2003 was low (less than 1%). After 2003, mortality was around 6–7% per year.

Individual level factors, including being male, low social class and relocation in the past two years were significantly associated with a higher incidence of mortality (Table 2). Area deprivation was also related to increased risk of mortality. A higher level of land use mix was associated with lower risk of five-year mortality with a potential decreasing trend (*p*=0.06). People living in the highest quartile of land use mix had a non-significant 20% higher risk of five-year mortality (HR: 0.79; 95% CI: 0.61, 1.01) compared to those in the lowest quartile after adjusting for individual level factors and area deprivation scores.

### 3.1 Interactions between age and land use mix

**Fig. 3:**
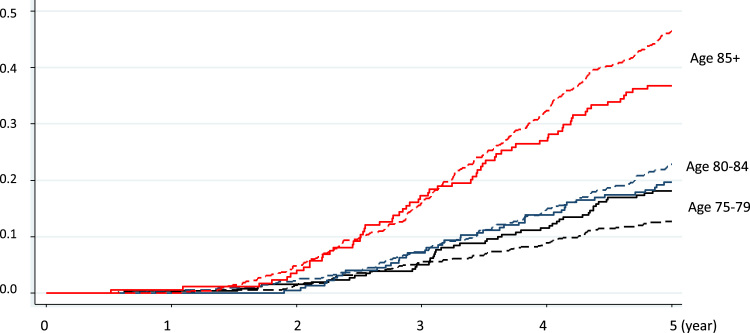
The cumulative incidence of five-year mortality by land use mix (the highest (dashed line) vs lowest quartile (solid line)) in three age groups (75–79, 80–84, 85+).

The cumulative incidence of five-year mortality by area level factors differed across younger and older age groups (Fig. 3). For the younger age group (75–79), the cumulative incidence of five-year mortality was higher in the highest quartile of land use mix but an opposite relationship was found in the oldest old (age 85 or above). Table 3 reports effect sizes estimated by age-stratified regression modelling. For those aged 75–79, living in the highest quartile of land use mix was associated with a non-significant 20% higher risk (HR: 1.22, 95% CI: 0.73, 2.04) of five-year mortality while in older age groups (80–84, 85+) high land use mix was associated with a lower risk of mortality. Particularly in the oldest old (age 85+), living in the highest quartile of land use mix was associated with 30% lower risk of five-year mortality (HR: 0.70; 95% CI: 0.49, 1.02) with a significant decreasing trend. In the 80–84 year old age group, although living in the highest quartile of land use mix was associated with a 40% lower risk of mortality (HR: 0.60; 95% CI: 0.37, 0.98), the trend across quartiles did not achieve statistical significance (*p*=0.08). The interaction terms between age groups and the highest quartile of land use mix achieved statistical significance (*p*=0.03).

### 3.2 Mediation effect of co-morbidity

Based on the diagrams presented in Fig. 1, Table 4 shows the mediation effect of co-morbidity on the associations between five-year mortality and land use mix. A competitive mediation effect was found in the overall population where both direct and indirect effects existed and pointed in opposite directions. For the younger age group (75–79), the effect of land use mix on mortality was mediated by co-morbidity. An indirect effect via co-morbidity shows a positive association between land use mix and co-morbidity (OR: 1.18; 95% CI: 1.08, 1.34) and increased risk of mortality in those with co-morbidity (HR: 1.28; 95% CI: 1.05, 1.50).

For the older age groups (80–84, 85+), mediation effects of land use mix were unclear. Although co-morbidity predicted a higher risk of five-year mortality across the whole cohort (HR: 1.28; 95% CI: 1.08, 1.50), the relationship between co-morbidity and land use mix was less clear in these older age groups compared to those aged 75–79. A higher level of land use mix was associated with reduced risk of mortality, with a strong direct effect particularly in the oldest old (HR: 0.87; 95% CI: 0.79, 0.99).

## 4 Discussion

### 4.1 Main findings

This study investigated the association between land use mix on subsequent mortality in a population aged 75 and over and further explored the potential modifying effect of age and mediating effect of co-morbidity. Differential effects were found across the age groups within the older population such that for the younger older people (75–79), living in the areas with high land use mix was associated with a non-significant 20% higher risk of mortality, a relationship mediated by co-morbidity. For older age groups (80–84, 85+), higher land use mix appeared to reduce five-year mortality.

### 4.2 Limitations

This study population included older people in large areas of England but cannot be seen as a random and representative sample. Since this is a year-10 follow-up interview, the problem of drop out after baseline leads to attrition effects. Individuals with lower levels of education and social class, poor health conditions and living in more deprived areas were less likely to respond to the year-10 interviews (Matthews et al., 2004). The variation for individual characteristics is possibly attenuated by dropout and death in this population.

Some participants could have changed their residence within the five years of follow-up but no information was available on these moves. The residential neighbourhoods of some individuals at death might be different from those recorded in the study. However, evidence suggests relatively few older people change their residence in the final year of their life (Fleming et al., 2010). A further limiting factor is that individuals living in care settings, who might have different interactions with community environments, cannot be identified separately in this study. Whilst the CFAS cohort was recruited randomly from several geographical areas with a high response rate, our analysis is drawn from participants in just four English centres and this may limit generalisability.

Since any effect of land use mix on mortality is likely to be the result of complex interactions between individuals and their environment, it is not possible to assess, in a cross sectional analysis, how large the time-lag might be between being exposed to a certain level of land use mix and mortality. The five-year follow-up period for mortality might therefore be too short. The neighbourhood environment of non-movers might change during follow-up, particularly in areas undergoing rapid development, yet the effect might not be detectable at the five-year point. The statistical “direct effect” we observed is unlikely to be causal. Some biological and behavioural factors such as physical activity and BMI might be strong mediators of the association and could provide potential explanations. Unfortunately, this information on these variables was not collected in the year-10 interview.

### 4.3 Land use mix and mortality: the younger and older old

High land use mix, related to better access to services and resources in local areas, has been suggested as an important factor to support active and healthy ageing (Croucher et al., 2005, Burton and Mitchell, 2006). The results of this study provide some support for a positive influence of land use mix on health in later life. In this population aged 75 or above, living in the highest quartile of land use mix was associated with over 20% lower risk of mortality than the lowest quartile.

We found that co-morbidity mediated the association between land use mix and mortality (Table 4) although not in the direction expected, as higher land use mix was associated with greater co-morbidity, particularly in the younger age group. The reasons for this warrant further investigation. They may be associated with migration in unwell individuals into mixed developments to be close to care. Indeed, a recent survey on ageing in place suggested that nearly 28% of individuals aged 75 or over changed their residences due to decline in their own or partner's health (Boldy et al., 2011). Alternatively, Saarloos et al. (2011) showed symptoms of depression were higher in areas with greater land use mix, and those authors suggest that this could be due to higher levels of incivility in mixed developments, a possible explanation for the unexpected mediation direction here.

We found differences in the direction of association between land use mix and mortality according to age, with evidence that residing in an area with more mixed land uses may be particularly protective for those aged 80 or over. Our mediation analysis showed that this association in the older age group was mostly “direct” from a statistical perspective. However, since land use mix is unlikely to directly cause death, there may be uncontrolled confounding or mediating factors which were not measured in this study. Living in areas with high land use mix might also reduce barriers to seeking help for emergency situations such as fall and injury. These may reduce mortality in very old age but was not mediated by co-morbidity. Using data from the Survey of Health, Ageing and Retirement in Europe (SHARE), Börsch-Supan et al. (2005) found that the reporting of chronic conditions was inversely associated with reported numbers of limitations in activity of daily living amongst the older old. Whilst the older old in our sample had more chronic conditions, it may therefore be that these people have better management and control of their conditions and are thus able to survive to very old age (Ofman et al., 2004). Living in areas with high land use mix might therefore particularly support these individuals living actively and independently and coping with basic needs in daily life.

### 4.4 Implications and future research directions

The findings of this study highlight potential different relationships between environment and health across stages within older age. Instead of considering older people as one group, policy planning should take note of such variation within older populations, and in particular the needs of the middle and oldest old cohorts. This observation is particularly relevant to the recent movement toward age-friendly environments, which have been advocated worldwide to create inclusive and supportive living environments for active ageing (World Health Organisation, 2007). Epidemiological evidence here shows that improving the mix of land uses in local areas may be a potential approach to reduce limitations in activity of daily life and support active ageing for these older age groups.

Current models of the components of the environment that may particularly strongly influence healthy ageing have largely been developed from qualitative research informed by a person-centred perspective and using relatively small numbers of older people (Menec et al., 2011, World Health Organisation, 2007). Population-based epidemiological cohorts often include older people living in diverse settings and can be used to test these models by quantifying environmental determinants of health in later life. To obtain a sufficient sample size and statistical power to detect the effect of place on the oldest old, linkage of existing longitudinal studies of ageing populations to nationwide databases of small area statistics, such as that undertaken here, will be a potentially fruitful approach. With the development of small area statistics, various measures of environmental context can be added to epidemiological cohorts and incorporated in future research in order to provide a nuanced understanding of ageing and place.

## Conflict of interest

We declare that we have no conflicts of interest.

## Figures and Tables

**Table 1:** The characteristics of the study population by age group (*N*, %).

		**Age 75–79**	**Age 80–84**	**Age 85+**
*N*		992	776	656
Sex	Men	419 (42.2)	305 (39.3)	229 (34.9)
	Women	573 (57.8)	471 (60.7)	427 (65.1)
Education	>9 years	404 (40.8)	313 (40.4)	249 (38.1)
(missing=6)	⩽9 years	586 (59.2)	462 (59.6)	404 (61.9)
Social class	Professional/managers	344 (34.8)	253 (33.1)	215 (33.0)
(missing=18)	Skilled non-manual	106 (10.7)	101 (13.2)	92 (14.1)
	Skilled manual	366 (37.0)	262 (34.3)	222 (34.1)
	Semiskilled/unskilled	174 (17.6)	148 (19.4)	123 (18.8)
Relocation in the past	No	872 (87.9)	658 (84.8)	530 (80.8)
two years	Yes	120 (12.1)	118 (15.2)	126 (19.2)
Number of chronic	0–1	653 (65.8)	463 (59.7)	359 (54.7)
conditions	2+	339 (34.2)	313 (40.3)	297 (45.3)
Year of death	2001–2002	14 (1.4)	13 (1.7)	29 (4.4)
	2003–2005	126 (12.7)	157 (20.2)	259 (39.5)
	2006–2008	156 (15.8)	147 (18.9)	176 (26.8)
	Survival	693 (70.1)	459 (59.1)	192 (29.3)

**Table 2:** The association between five-year mortality, individual and area level factors.

		**Model 1**	**Model 2**
HR (95% CI)	HR (95% CI)
*Individual level*			
Age groups	75–79	1.00	1.00
	80–84	1.61 (1.29, 2.02)	1.58 (1.26, 1.98)
	85+	3.77 (3.08, 4.62)	3.77 (3.07, 4.62)
Gender	Women	1.00	1.00
	Men	1.20 (1.02, 1.41)	1.32 (1.12, 1.56)
Education	>9 years	1.00	1.00
	⩽9 years	1.17 (0.99, 1.38)	0.92 (0.76, 1.11)
Social class	Professional/managers	1.00	1.00
	Skilled non-manual	1.52 (1.16, 1.98)	1.43 (1.09, 1.87)
	Skilled manual	1.33 (1.08, 1.63)	1.25 (1.00, 1.57)
	Semiskilled/unskilled	1.66 (1.32, 2.08)	1.57 (1.22, 2.00)
Relocation in the	No	1.00	1.00
past two years	Yes	1.56 (1.28, 1.91)	1.38 (1.13, 1.69)
			
*Area level*			
Deprivation score		1.01 (1.00, 1.01)	1.01 (1.00, 1.01)
Land use mix	Q1 (lowest)	1.00	1.00
	Q2	1.01 (0.80, 1.26)	0.95 (0.75, 1.21)
	Q3	1.03 (0.92, 1.29)	0.91 (0.72, 1.16)
	Q4 (highest)	0.95 (0.76, 1.19)	0.79 (0.61, 1.01)
*p*-value (test for trend)		0.68	0.06

**Model 1**: unadjusted model; **Model 2**: adjusted for individual level factors (age group, sex, education, social class and relocation in the past two years) and deprivation score.

**Table 3:** The association between mortality, area deprivation and land use mix by three age groups (75–79, 80–84 and 85+).

**Age group**		**75–79**	**80–84**	**85+**
		HR (95% CI)	HR (95% CI)	HR (95% CI)
Land use mix	Q1 (lowest)	1.00	1.00	1.00
	Q2	0.90 (0.54, 1.51)	0.78 (0.49, 1.24)	1.09 (0.79, 1.51)
	Q3	0.97 (0.58, 1.61)	0.92 (0.59, 1.45)	0.88 (0.63, 1.24)
	Q4 (highest)	1.22 (0.73, 2.04)	0.60 (0.37, 0.98)	0.70 (0.49, 1.02)
*p*-value (test for trend)		0.38	0.08	0.03

All estimates were adjusted for sex, education, social class and relocation in the past two years and deprivation score. The interaction terms between age groups and highest quartile of land use mix achieved statistical significance (*p*=0.03).

**Table 4:** Mediation effects of co-morbidity on the association between five-year mortality and land use mix (trends across quartile) by three age groups.

	**Overall population**	**Age 75–79**	**Age 80–84**	**Age 85+**
*a*	1.10 (1.02, 1.19)	1.18 (1.03, 1.34)	1.09 (0.94, 1.26)	1.05 (0.90, 1.22)
*b*	1.28 (1.08, 1.50)	1.28 (1.08, 1.50)	1.49 (1.10, 2.02)	0.95 (0.75, 1.20)
*c*	0.92 (0.85, 1.00)	1.05 (0.88, 1.24)	0.87 (0.75, 1.01)	0.88 (0.79, 0.99)
Total effect	0.93 (1.02, 1.19)	1.08 (0.97, 1.27)	0.87 (0.75, 1.02)	0.88 (0.79, 0.99)
Direct/indirect effect	Competitive	Indirect	Unclear	Direct

The effect of *path a* was odds ratio (95% CI), estimated by logistic regression. The effects of *path b* and *c* were hazard ratios, estimated by Cox proportional hazard regression.
